# Effects of saline-alkali stress on cotton growth and physiochemical expression with cascading effects on aphid abundance

**DOI:** 10.3389/fpls.2024.1459654

**Published:** 2024-10-08

**Authors:** Yu Gao, Bing Liu, Hongyi Wei, Yanhui Lu

**Affiliations:** ^1^ Ministry of Education Key Laboratory of Crop Physiology, Ecology and Genetic Breeding, Jiangxi Agricultural University, Nanchang, China; ^2^ State Key Laboratory for Biology of Plant Diseases and Insect Pests, Institute of Plant Protection, Chinese Academy of Agricultural Sciences, Beijing, China; ^3^ Western Agricultural Research Center, Chinese Academy of Agricultural Sciences, Changji, China

**Keywords:** aphid pest, bottom-up, environment stress, interaction, salinity, alkalinity

## Abstract

**Introduction:**

Environmental stresses, such as soil salinity or alkalinity, usually affect crop growth and secondary plant metabolism, with follow on effects on foliar-feeding insects. Nevertheless, the underlying mechanism of how saline-alkali stress affects the key cotton pest *Aphis gossypii* Glover is poorly understood.

**Methods:**

In this study, we first considered effects of three types of saline-alkali stress (i.e., salinity alone, alkalinity alone – both at different concentration – and their mixed effects) on cotton plants. We then measured impacts of stress on (1) above and below plant growth traits (e.g., plant height, leaf area, root volume), (2) levels of nutrients and secondary metabolites in cotton leaves, and (3) feeding behavior, life-table parameters, and population growth of *A. gossypii*. We then used a path analysis to evaluate cascading effects of changes in plant growth (due to stress) and changes in levels of nutrients or secondary metabolites on growth of individual cotton aphids and aphid populations.

**Results:**

We found either salinity or alkalinity stresses significantly reduced cotton growth, increased the content of tannin, soluble sugars, and proline in the leaves, and suppressed aphid growth and development, (including longevity, fecundity, and intrinsic rate of increase) and aphid population growth. Alkalinity had stronger effects on these traits than did salinity.

**Discussion:**

This work provides insights into the bottom-up interaction mechanism by which these environmental stresses mediate aphid infestation levels in the cotton agricultural ecosystem.

## Graphical Abstract

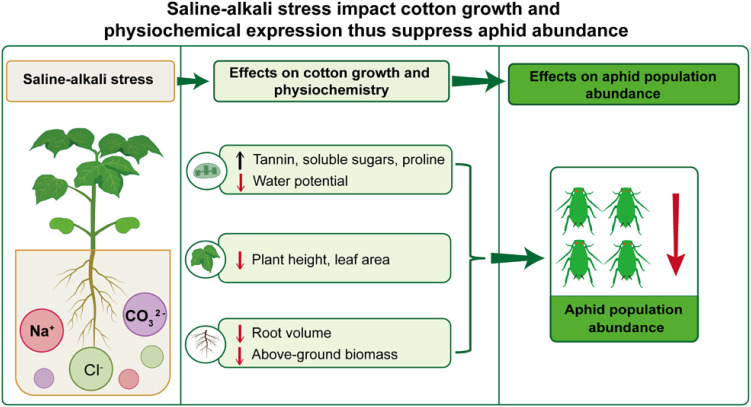

## 1 Introduction

Soil salinity is a major abiotic stress on crops in agricultural systems, and it is predicted that due to global warming and other factors, more than 50% of arable land will be affected by salt stress by 2050 (Zhang et al., 2023). Neutral salts (i.e., NaCl and Na_2_SO_4_) and alkali salts (i.e., NaHCO_3_ and Na_2_CO_3_) are two distinct types of abiotic stress. Neutral salts inhibit the growth and metabolic processes of crops and cause osmotic stress, nutrient imbalance, or even death (Guo et al., 2019; Hou et al., 2023). By comparison, high pH from alkali stress around roots can destroy cell membrane integrity and hinder the absorption of ions through the precipitation of Ca^2+^, Mg^2+^, and HPO_3_^−^ (Fang et al., 2021). This damage leads to the disruption of ion balance and severely impairs the root system (Guo et al., 2016). Several studies have demonstrated that the adverse effects of alkali stress were greater than those of neutral salt stress. For instance, Yang et al. (2008) found the destructive effects of alkalinity treatment on the growth and photosynthesis of wheat to be more severe than those of salinity. Similarly, alkali stress inhibited the growth of *Leymus chinensis* (Trin.). Tzvelev more than salt stress, causing a significant reduction of plant height and shoot dry weight under both mild and severe alkali stresses (Yan et al., 2023). In canola (*Brassica napus* L.), alkali salts (Na_2_CO_3_) inhibited seed germination and seedling growth significantly more than a neutral salt (NaCl) treatment (Wang et al., 2022).

Cotton is an economic key agricultural crop worldwide (Xu et al., 2020). In 2023, China’s cotton production was 5.618 million tons, of which 84.9% and 90.9% of planting area and yield were in Xinjiang province, respectively (National Bureau of Statistic, 2023), which has the widest distribution of saline–alkali soils in China. The soil pH is generally above 8.5, and soil salinization and alkalization often occur simultaneously (Guo et al., 2019). Although cotton is classified as a salt-tolerant crop, it can still be injured by excessive salt levels (Dai et al., 2014). Guo et al. (2022) found that salt-treated and alkali-treated cotton plants showed declines of 51.8% and 53.0% in biomass, and salt or alkali stress altered levels of organic acids, amino acids, and sugars in leaves and roots of seedlings. Salt stress of cotton seedlings inhibited root development and plant growth, resulting in reduced plant height; reduced total root length, surface area, and volume; and reduction in taproot length and surface area (Duan et al., 2022).

*Aphis gossypii* Glover (Hemiptera: Aphididae) is widespread in cotton fields in Xinjiang, China. It frequently causes severe damage, reducing productivity (Li et al., 2018; Yi et al., 2023). Growth and development of cotton aphids mainly rely on nutritional sources in the phloem vessels in leaves and stems. The levels of such nutrients and of defense metabolites change under salt or alkali stress, altering aphid performance (Inbar et al., 2001; Denno et al., 2002; Elias, 2014). For these stresses, most studies have focused on the effects of salinity, whose effects on host plant quality can have various effects on associated herbivores. For example, Han et al. (2016) found that the treatment of “optimal water + high salinity” increased the development rate of the moth *Tuta absoluta* (Meyrick) on tomato without lowering pupal mass. Polack et al. (2011) showed that aphid density was higher on sweet pepper irrigated with high conductivity solutions, whereas Eichele-Nelson et al. (2018) found that as salinity increased, both population size and fecundity of soybean aphid increased on soybean plants and that aphids lived longer and produced more offspring under high-salinity conditions. However, Ma et al. (2021) found that salt stress increased accumulation of the gossypol and tannin in cotton seedlings, which inhibited the population growth of cotton spider mites. Also, Zhang et al. (2015) found that the population density of cotton aphids fed cotton stressed with 150 mM or 200 mM NaCl was strongly reduced (65%–100%) after 1 to 2 weeks. Similarly, Sienkiewicz-Paderewska et al. (2017) reported that the Cl content of leaves of Crimean linden was raised from 1% to 2%, the abundance of lime aphids (*Eucallipterus tiliae* L.) by 36%.

In this study, we subjected cotton seedlings to varying degrees of salt, alkali, or combined stress under hydroponic cultivation to evaluate the joint and separate effects of salt and alkali stress on the growth and physiochemical properties of cotton plants, as well as aphid life-table parameters and population dynamics. Our specific aims were to assess (1) how salt or alkali stress affected the growth, development, and physiological or biochemical properties of cotton plants; (2) how the impacts of salt-alkali stress on plants further affected the fitness of individual aphids and their feeding behavior; and (3) how salt or alkali stresses affected the population dynamics of cotton aphids.

## 2 Methods

### 2.1 Plant and insect rearing

Cotton seeds (*Gossypium hirsutum* L., cultivar “Zhongmian 49”) were sown in plastic pots (12 cm dia, 10 cm height) containing peat, vermiculite, and field soil (volume ratio: 6:1:1) and grown in a greenhouse at 28°C~30°C, 50 ± 5% RH, and 16:8 (L: D) h photoperiod. When most of cotyledons fully expanded, cotton seedlings were separately transferred into a 1/2 Hoagland’s hydroponic culture solution in a new jar (11 cm dia, 13.8 cm height) and after 7 days were moved to full Hoagland’s hydroponic culture.

The *A. gossypii* aphids for our colony were collected from cotton fields in Korla Experimental Station, Institute of Plant Protection, Chinese Academy of Agricultural Sciences (CAAS; 41.45° N, 85.48° E) (Korla, Xinjiang Province, China), in 2019 and were maintained on cotton seedlings with five to six leaves for multiple generations in a growth chamber at 26 ± 1°C, 50 ± 5% RH, and 16:8 (L:D) h photoperiod at the Langfang Experimental Station, Institute of Plant Protection, Chinese Academy of Agricultural Sciences (CAAS; 39.53° N, 116.70° E) (Langfang, Hebei Province, China).

### 2.2 Salt and alkali stress treatments

As treatments, we investigated two common types of soil salinization, based on the salt content and pH of most alkali soils in Xinjiang, i.e., chloride (NaCl) and carbonate (Na_2_CO_3_). Four treatments were two NaCl concentrations (75 mM and 150 mM) and two Na_2_CO_3_ concentrations (5 mM and 12 mM), whereas 5 mM Na_2_CO_3_ and 12 mM Na_2_CO_3_ corresponded to a pH of 8.7 and 9.5, respectively. Joint saline-alkali treatments were developed by co-stressing plants with both NaCl and Na_2_CO_3_, each at two levels. Non-salt, non-alkali stress was set as the control condition. In summary, there were two levels of each of saline stress (Salinity_middle [Sm], Salinity_high [Sh]) and alkali stress (Alkalinity_middle [Am], Alkalinity_high [Ah]). For mixed stress treatments, we examined three combinations: Sm_Am, Sm_Ah, Sh_Am) (for treatment details see **Supplementary Table S1**).

To apply treatments, when most seedlings were in the three-leaf stage, healthy plants of uniform sizes were selected and treated with the nutritional solution amended with NaCl or Na_2_CO_3_. To acclimatize plants to NaCl or Na_2_CO_3_ stress, we employed a stepwise increase in the concentration of salt or alkali. The final concentration was reached by following this procedure: On the first day of stress, 25 mM and 50 mM NaCl (for the Salinity_middle and Salinity_high treatments, respectively) were added to the normal nutrient solution. On the second day, the nutrient solution was replaced with 50 mM and 100 mM NaCl, and on the third day, it was replaced by 75 mM and 150 mM NaCl. Similar for Na_2_CO_3_, 1 mM and 4 mM Na_2_CO_3_ (for Alkalinity_middle and Alkalinity_high treatment) were added on the first day. The solution was then replaced with 3 mM and 8 mM Na_2_CO_3_ on the second day, and with 5 mM and 12 mM Na_2_CO_3_ on the third day. The saline-alkali stress treatments involved a mixture of NaCl and Na_2_CO_3_ in the nutrient solution. After reaching the final concentration, the nutrient solution was replaced every 5 days. The stock solution was maintained at room temperature.

### 2.3 Measurement of plant growth traits

#### 2.3.1 Plant height and leaf area

For all treatments, we measured plant height and leaf area once each week after the initiation of salt or alkali stress for five plants per treatment. Plant height was defined as the distance from the cotyledon node to the top of the cotton plant. Leaf area was measured by removing three 1-cm^2^ holes with a leaf punch, which were then weighed and dried at 70°C until a constant mass was achieved. The leaf disc weight divided by their total area was the specific leaf weight. The total leaf area was then calculated by dividing the total leaf weight of the whole plant by the specific leaf weight, as calculated above (Junior and Kawakami, 2013).

#### 2.3.2 Measurement of plant biomass

To determine the aboveground biomass of cotton, the weight of the whole plant along with roots, stems, and leaves were measured using a weighing balance 28 days after the initiation of salt or alkali stress. We measured the aboveground biomass for five replications, using one cotton seedling per replication.

#### 2.3.3 Root characteristics

The roots of cotton seedlings were scanned using an Epson scanner after 28 days of salt or alkali stress. The root volume was measured using WinRhizo (V2.0) image analysis software. We measured the root volume for five replications, using one cotton seedling per replication.

### 2.4 Measurement of plant physiological and biochemical traits

After plants were subjected to salt or alkali stress for 20 days, we measured the physiochemistry (i.e., the contents of tannin, soluble sugars, proline, and water potential) of cotton leaf (all in seedling period). Three plants were chosen as three replications for each treatment. The third leaf on the main stem from the top of each plant was removed and quickly frozen in liquid nitrogen. It was then stored at −80°C for measurement of levels of tannin, soluble sugars, and proline. Tannin was measured using Folin’s assay (Guinn and Eidenbock, 1982), and soluble sugars were measured using the anthrone reagent method (Yemm and Willis, 1954). Similarly, for each treatment, three new plants were chosen to detect the leaf (the third leaf on the main stem from the top) water potential using a pressure chamber (Zhejiang Top Cloud-Agri Technology Co., Ltd., TP-PW-II, China).

### 2.5 Effect of salt-alkali stress on feeding behavior of *A. gossypii*

The feeding behavior of *A. gossypii* was analyzed using the electrical penetration graph (EPG) technique (Tjallingii, 1985, 1988), on a Giga-8 complete system (manufactured by Wageningen University, Wageningen, The Netherlands) according to the method by Tjallingii and Esch (Tjallingii and Esch, 1993; Walker et al., 2024). Newly emerged apterous adult aphids (<12 h old) were randomly selected and starved for 1 h before recording their behaviors; meanwhile, a gold wire (2 cm length, 18.5 μm diameter) was attached to the aphid dorsum using water-soluble conductive silver glue. Then, the opposite end of the gold wire was glued to a copper wire (2 cm length, 2 mm diameter) attached to a brass pin that was inserted into the EPG probe as the insect electrode. A wooden stick was then secured vertically with adhesive tape on the jar, which was holding nutrient solution and a cotton seedling after 20 days of salt or alkali stress. A piece of stiff paper, larger than the cotton leaf in size, was attached onto the stick at the same height as the cotton leaf. We then turned over the third leaf from the top of each plant cotton and fixed it onto the piece of paper, and adjusted the insect electrode so that the aphids could rest on the underside of the leaf. Another copper electrode (10 cm length, 2 mm diameter) was inserted into the nutrient solution. After wiring of aphids to the recording device was completed, cotton seedlings were placed in a Faraday cage, and aphid feeding waveforms were simultaneously recorded on eight plants of each different stress treatment after plants were placed at random in a Faraday cage. Aphids were then allowed to feed for 8 h, and aphid feeding behavior was recorded while being held at 26 ± 1°C, 50 ± 5% RH. There were 15 replicates (one plant with one aphid being a replicate) for each treatment; all data from the test insects were collected and annotated using Stylet+ software (Wageningen Agricultural University, Wageningen, The Netherlands). We imported the ANA file exported from the Stylet+ software into Excel 2019, and then we classified the data by the filters in Excel and manually calculated all the EPG parameters. Waveform patterns were scored and assigned to previously described categories (Garzo et al., 2016; MaChado Assefh et al., 2014): (1) non-probing/penetration (Np), which represents non-probing behavior (no stylet contact with the leaf tissue); (2) initiation of stylet penetration of leaf tissue (C), which represents the intercellular apoplastic stylet pathway where the insects show a cyclic activity of mechanical stylet penetration and secretion of saliva; (3) salivation into phloem sieve elements at the beginning of the phloem phase (E1); (4) passive phloem sap uptake from the sieve element (E2); (5) short bouts of intracellular penetration (Pd.); (6) active intake of xylem sap (G).

### 2.6 Treatment effects on the growth and development of individual aphids and construction of life-tables

To obtain data for life table analysis of aphids reared on plants in different stress treatments, we first confined three newly emerged apterous adult aphids on the third leaf of each test plant (7 days after the initiation of salt or alkali stress) in a nylon mesh bag. After 24 h, these adult aphids were removed from the leaves with a small artist brush, leaving one newborn nymph on each plant within each nylon mesh bag. Plants with aphids were held in a growth chamber at 26 ± 1°C, 50 ± 5% RH, 16:8 (L: D) h photoperiod. We checked the nymphs daily and recorded their life stage. After a test nymph became an adult, we recorded the number of born offspring daily, removing them each day. This continued until all the original aphids died. Each of the 30 seedling per treatment received one nymph (= 30 replications/treatment) that were followed in this manner.

Age-stage, two sex life tables (Chi et al., 2020) were used to analyze the life history data for cotton aphids subject to our experimental treatments using “TWOSEX-MS Chart for the Windows operating system” software, available online at http://140.120.197.173/ecology/products.htm. Age-specific survival rate (*l_x_*), survival rate (*s_xj_*; where *x* = insect age and *j* = life stage), mean generation time (*T*), fecundity (*m_x_*), net reproductive rate (*R*_0_), intrinsic rate of increase (*r*_m_), and finite rate of increase (*λ*) were calculated as follows:


(1) $$\text{age-specific survival rate: }l_{x}=\sum_{j=1}^{m}s_{xj}$$

(2) $$\text{age-stage specific survival rate: }m_{x}=\frac{\sum_{j=1}^{m}S_{xj}f_{xj}}{\sum_{j=1}^{m}S_{xj}}$$

(3) $$\text{intrinsic rate of increase: }\sum_{x=0}^{\infty }{\text{e}}^{-r\left(x+1\right)}l_{x}m_{x}=1$$

(4) $$\text{finite rate of increase: }\sum_{x=0}^{\infty }\left(\lambda^{-\left(x+1\right)}\sum_{j=1}^{m}f_{x_{j}}s_{xj}\right)=1$$

(5) $$\text{net reproductive rate: }R_{0}=\sum_{x=0}^{\infty }l_{x}m_{x}$$

(6) $$\text{mean generation time: }T=\frac{\ln R_{0}}{r}$$

### 2.7 Effects of salt or alkali stress on aphid population dynamics

Seven days after the initiation of salt or alkali stress, mesh-nylon cages were placed over 15 individual cotton seedlings (i.e., our replicates) and five newly emerged apterous adult aphids were placed on the third leaf (on the main stem from the top to down) of each plant. One day later, these adult aphids were removed with a small artist brush leaving five newborn nymphs on each plant. Infested plants were held in a growth chamber at 26 ± 1°C, 50 ± 5% RH, 16:8 (L: D) h photoperiod, and we recorded the number of aphids (nymphs and adults) on the whole plant every 5 days thereafter until the experiment ended on day 55.

### 2.8 Cascading effects of salt or alkali stress on plant and aphid growth

Based on the above observations, we assessed the direct effects of salt or alkali stress on plant growth, plant physiochemistry, aphid life-table parameters, and aphid population density using principal component analysis (PCA). Path analysis (structural equation model, SEM) was then used to identify cascading effects among significant variables.

### 2.9 Statistical analysis

Plant growth indices (i.e., height, leaf area) that were repeatedly measured during 1~6 weeks were analyzed by a linear mixed effect model (LMM) with the REML (restricted maximum likelihood) estimation method (Zuur et al., 2013). In the model, treatment, detection date, and their interaction were the fixed effects, and replication was the random effect. For other plant growth indices that were measured only once (i.e., aboveground fresh weight, root volume), measured 4 weeks after salt or alkali stress, they were analyzed using one-way analysis of variance (ANOVA), with Tukey’s HSD to compare treatment means. For plant biochemical properties and the parameters of the aphid EPG experiment, ANOVAs were also performed, and Tukey’s HSD was used to compare treatment means. The life-table parameters of *A. gossypii* were analyzed using “TWOSEX-MS Chart for the Windows operating system” software (Chi, 1988), available online at http://140.120.197.173/ecology/products.htm. LMMs were carried out with the “lmerTest” package (Kuznetsova et al., 2017), ANOVAs were run using the “stats” package, and multiple comparisons were made by the “emmeans” package (Lenth, 2022) of R 4.2.1 software (R Development Core Team, 2022).

For the population data on *A. gossypii*, we employed a generalized linear mixed effect model (GLMM) with negative binomial residual distribution (Zuur et al., 2013) to analyze the variation of population abundance of *A. gossypii* under different salt or alkali stress. In the GLMM, aphid abundance was the response variable; the fixed effects were treatments (a total of eight groups of salt, alkali, and their mixed treatments), survey time (the days after aphid colonization), and their interaction; and replication (15 replications for each treatment) was the random effect. The significance of fixed effects (*P* values of type II Wald χ^2^ tests) was tested using the ‘Anova’ function from the “car” package (Fox and Weisberg, 2019). GLMM analysis was performed with the “glmmTMB” package (Brooks et al., 2017).

Principal component analysis (PCA) was used to illustrate relationships between different variables under salt or alkali stress. The plant growth indicators included plant height, leaf area, aboveground fresh weight, and root volume. The physiochemical properties included leaf water potential, the content of tannin, soluble sugars, and proline. The aphid life-table parameters included *R*_0_, *λ*, *r*_m_, and *T*. The mean abundance was used to indicate the population dynamic across the whole detection period. PCA was performed by using the “vegan” package (Oksanen et al., 2022).

**Figure 1:**
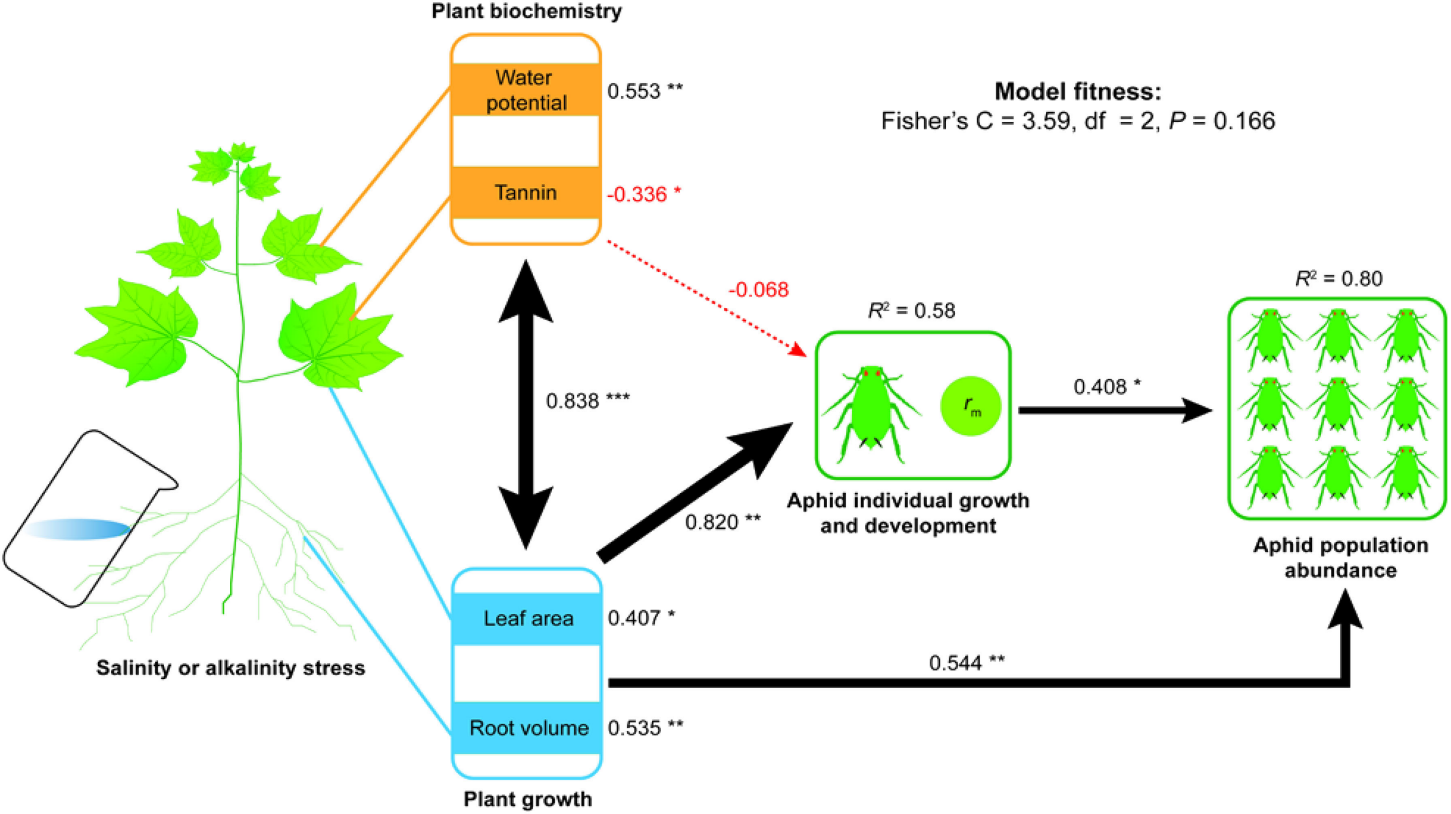
Cascade effects of saline–alkali stress driven plant growth and physiochemical properties on aphid development or the population abundance (model details see **Supplementary Table S6**). Plant growth traits include leaf area and root volume; plant physiochemical properties include leaf water potential and tannin content. Standardized coefficients explain that the effect size is shown for each path and is scaled as line widths. *R* ^2^ values indicate the explanatory ability relative to the total variance for each response variable in the model. Black and red lines indicate positive and negative relationships, with solid lines representing statistically significant effects (*P< 0.05, **P< 0.01, ***P< 0.001) and dotted line showing non-significant effects.

Path analysis with structural equation modeling (SEM) was used to evaluate the direct and indirect effects of salinity–cotton–pest network interactions. Variables used in the path analysis were selected based on previous ANOVA, GLMM analysis, and PCA. In SEM, in order to clarify the effects of different types of predictors, two combinate variables were structured to represent plant growth indicators (including two separate factors, leaf area, and root volume) and plant physiochemical properties (including of water potential, the content of tannin in cotton leaf), respectively. We chose intrinsic the rate of increase (*r*_m_) to represent the aphid individual life table parameters, and the mean abundance of the aphid populations across the whole detection period was the response variable. SEM analyses were executed in two steps. First, we allowed all effects and paths into the model; second, we removed the non-significant paths to optimize the analysis based on the Akaike information criterion (where smaller AIC values are better), and the final results and path diagrams were presented in **Supplementary Table S6** and **Figure 1**. In the SEM analysis, the standardized path coefficient indicated the effect size of different predictors on each response variable, and *R* ^2^ represented the explanatory ability on a given response variable. The global goodness of fit with Fisher’s C statistic was tested to determine model fitness (*P* > 0.05 indicated the model was fitted well). Path analyses were carried out using “psem” function in “piecewiseSEM” package (Lefcheck, 2016). All statistical analyses were performed by using R 4.2.1 software (R Development Core Team, 2022).

## 3 Results

### 3.1 The effect of salt or alkali stress on plant growth traits

**Figure 2:**
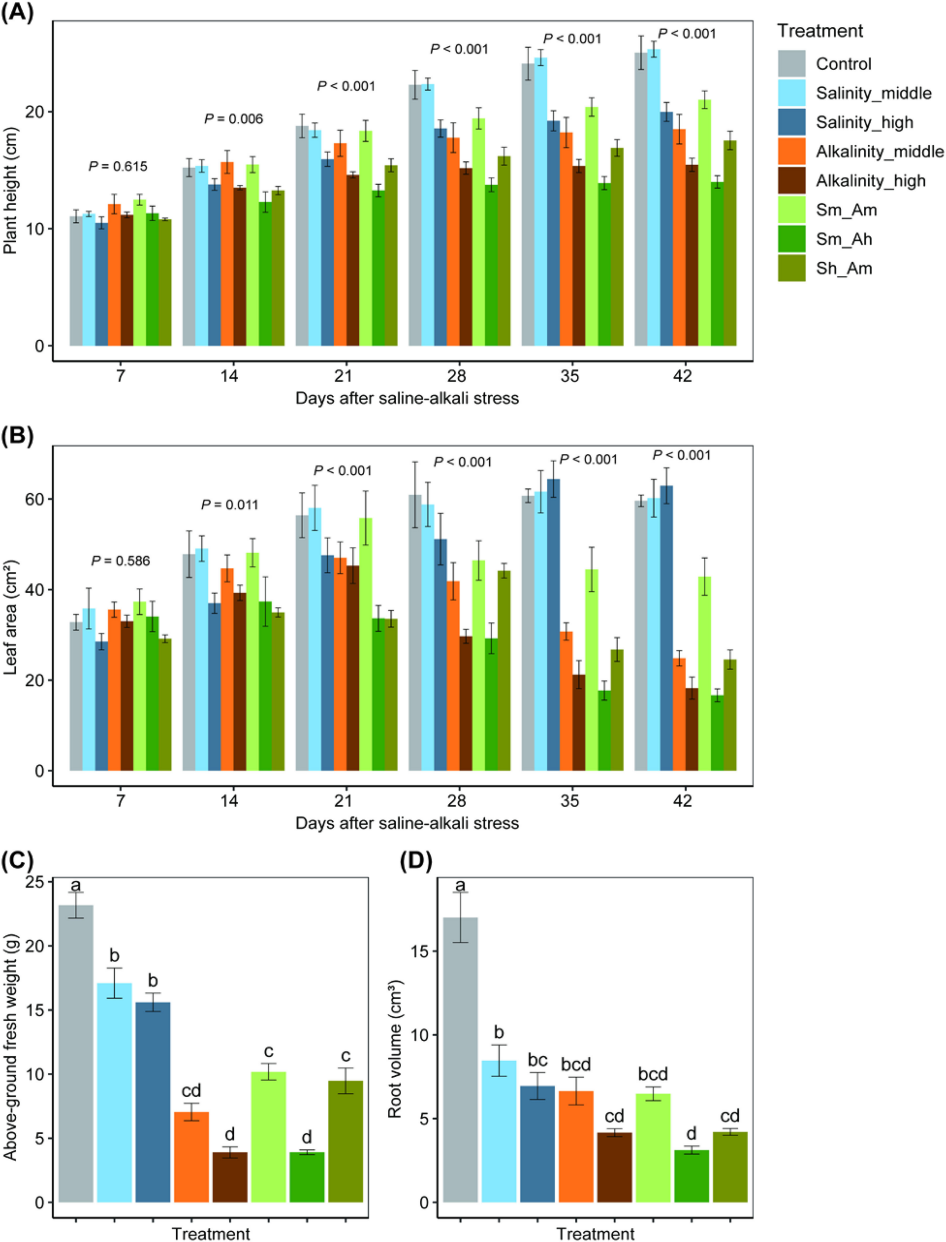
Effects on the plant height **(A)**, leaf area **(B)**, aboveground weight **(C)**, and root volume **(D)** of cotton seedlings under different concentrations of salt or alkali stress. Bars show means ± SE. Values with different lowercase letters are significantly different at *P*< 0.05.

Plant height. LMM analysis showed that salt or alkali stress significantly affected cotton growth (**Figure 2**). A significant difference was found in plant height among treatments (χ^2^ = 419.40, df = 7, *P*< 0.001), and these differences changed over cotton growth times (7~42 days after stress deployment, χ^2^ = 729.40, df = 5, *P*< 0.001, **Figure 2A**, **Supplementary Table S4**). During the first 7 days after initiation of salt stress, there was no significant difference in plant height among treatments (*F*_7,188_ = 0.77, *P* = 0.615); however, significant differences existed at 14, 21, 28, 35, and 42 days (**Figure 2A**). For instance, at 42 days after stress deployment, plant heights of the Salinity_high, Alkalinity_middle, and Alkalinity_high treatments were 20.2%, 26.1%, and 38.3% lower than the control treatment, respectively, whereas the plant heights of the mixed salinity-alkalinity treatments (Sm_Am, Sm_Ah and Sh_Am) were 16.1%, 44.1%, and 30.0% lower than the control (*F*_7,188_ = 30.52, *P*< 0.001). However, no significant difference was found between control and the Salinity_middle treatment.

Leaf area. A significant difference in leaf area existed among treatments (χ^2^ = 366.41, df = 7, *P*< 0.001), and differences changed over cotton growth times (7~42 days after stress deployment, χ^2^ = 80.06, df = 5, *P*< 0.001, **Figure 2B**; **Supplementary Table S4**). As with plant height, 7 days after initiation of stress, there was no significant effect of salt or alkali stress on leaf area (*F*_7,188_ = 0.80, *P* = 0.586); however, significant differences existed at 14, 21, 28, 35, and 42 days (**Figure 2B**). For instance, on the 42 days, the leaf areas of the Alkalinity_middle and Alkalinity_high treatments were 58.3% and 69.4% smaller than the control, whereas the leaf area was also significantly smaller (by 28.1%, 72.1%, and 58.8%) under the salinity–alkalinity treatments (Sm_Am, Sm_Ah and Sh_Am), respectively (*F*_7,188_ = 32.74, *P*< 0.001). With increasing salinity level, there was no significant further reduction in leaf area.

Aboveground fresh weight. On the 28 days after initiation of the salt or alkali stress, all salinity and alkalinity treatments (or their mixed treatments) had significant negative effects on fresh weight compared with the control (*F*_7,32_ = 74.43, *P<* 0.001), and effects of alkalinity were larger than those of the salinity treatments (**Figure 2C**).

Root volume. As aboveground fresh weight, root growth (indicated by the root volume) was also significantly reduced by the salt or alkali treatments compared with the control (*F*_7,32_ = 32.06, *P<* 0.001); however, no significant differences were found between salinity and alkalinity mixed treatments (**Figure 2D**).

These results indicated that plant growth traits of cotton were significantly and negatively affected by salt or alkali stress, especially for the alkalinity and the mixed treatments.

### 3.2 Effects of salt or alkali stress on plant physiochemistry

**Figure 3:**
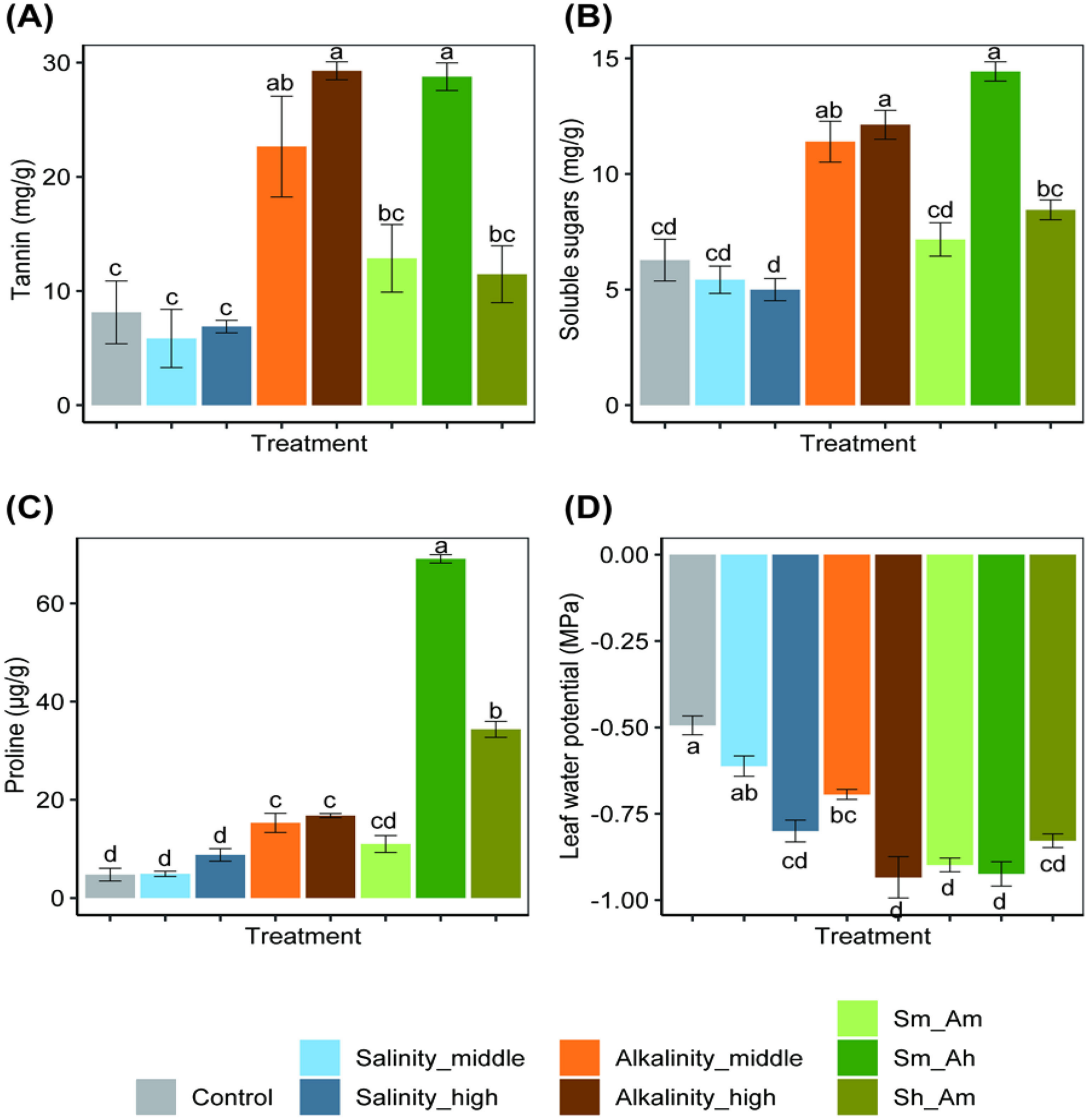
Effects on levels of tannin **(A)**, soluble sugars **(B)**, proline **(C)**, and leaf water potential **(D)** of cotton seedlings under different concentrations of salt or alkali stress. Bars show means ± SE. Values with different lowercase letters are significantly different at *P*< 0.05.

Salt or alkali stress significantly affected the physiochemistry of plant, including effects on levels of tannin, soluble sugars, proline, and the water potential of cotton leaves (**Figure 3**).

Tannin. After 20 days, the tannin content of leaves was significant varied among treatments (*F*_7,16_ = 14.87, *P*< 0.001). Under alkali stress, tannin contents of the Alkalinity_middle and Alkalinity_high treatments were 2.8 and 3.6 times higher than those of the control. Under combined saline–alkali stress, tannin levels in the Sm_Am, Sm_Ah, and Sh_Am treatments were 1.6, 3.5, and 1.4 times higher than in the control, respectively. The middle and high levels of the saline stress had no significant impacts on tannin content; meanwhile, no significant differences were observed in the case of Sm_Am and Sh_Am (**Figure 3A**).

Soluble sugars. Salt or alkali stress affected the level of soluble sugars (*F*_7,16_ = 28.05, *P<* 0.001, **Figure 3B**). Compared with salinity treatment, alkalinity had a significant effect on levels of soluble sugars, being 1.82 and 1.93 times higher in the Alkalinity_middle and Alkalinity_high treatments than in the control. For the mixed treatment with high alkalinity (Sm_Ah), there was a 1.29-fold increase in soluble sugar compared with the control, but the salinity treatments (middle and high salinity) did not differ from the control.

Proline. Salt or alkali stress significantly affected the proline content of cotton leaves (*F*_7,16_ = 271.70, *P<* 0.001, **Figure 3C**). As the alkali concentration increased, the proline level also significantly increased, being 2.2 and 2.5 times higher in the Alkalinity_middle and Alkalinity_high treatments than in the control. Under combined saline–alkali stress, the proline contents in the Sm_Ah and Sh_Am treatments was 14.5 and 7.2 times those in the control, with significant differences among the treatments. However, salinity treatments (both middle and high concentrations) had no significant impacts on proline content.

Water potential. Salt or alkali stress significantly reduced the water potential of cotton leaves (*F*_7,32_ = 24.19, *P<* 0.001, **Figure 3D**). Compared with the control, the leaf water potential decreased by 24.5% and 63.3% in the middle- and high-salinity treatments, and the Alkalinity_middle and Alkalinity_high treatments resulted in a decrease of 40.8% and 89.8%, respectively. Similarly, the combined treatments (Sm_Am, Sm_Ah, and Sh_Am) showed a decrease of 83.7%, 87.8%, and 69.4%, respectively. These results indicate that under various salt and alkali stress treatments, cotton seedlings experienced different levels of water deficiency, which negatively affected their growth.

### 3.3 Effects of salt and alkali stress on feeding behavior responses of aphids

Electrical penetration graph (EPG) detection showed that salt or alkali stress affected the feeding behavior of cotton aphids (**Supplementary Tables S2**, **S3**). There were significantly differences in the number of C waves (intercellular apoplastic stylet pathway) (*F*_7,112_ = 3.75, *P* = 0.001), E1 waves (phloem salivation) (*F*_7,112_ = 3.71, *P* = 0.001), E2 waves (phloem ingestion) (*F*_7,112_ = 3.80, *P* = 0.001), and Np waves (non-probing/penetration) (*F*_7,112_ = 6.07, *P<* 0.001) for aphids feeding on different treatments. Meanwhile, there were significantly differences in the total duration of C waves (*F*_7,112_ = 4.53, *P* = 0.002), E2 waves (*F*_7,112_ = 3.14, *P* = 0.005), Pd waves (short bouts of intracellular penetration) (*F*_7,112_ = 2.68, *P* = 0.013), Np waves (*F*_7,112_ = 6.97, *P<* 0.001), and G waves (active intake of xylem sap) (*F*_7,112_ = 3.18, *P* = 0.005). For the time to first E1 and E2, there were also significantly differences among the treatments (*F*_7,112_ = 2.51, *P* = 0.02), (*F*_7,112_ = 9.04, *P<* 0.001).

With increasing salt stress, the number of C, E1, and E2 waves as well as the total duration of Pd increased significantly under the Salinity_high treatment compared with the control. With increasing alkali concentration, the number of C waves and the total duration of C and Pd increased significantly, whereas the total duration of E2 significantly decreased under Alkalinity_middle treatment compared with the control; meanwhile, the number of E1 and E2 waves and the total duration of Pd increased significantly under Alkalinity_high treatments. Under combined saline–alkali stress, compared with the control treatment, the number of C and Np waves and the total duration of Pd waves increased significantly under Sm_Am, whereas the Sm_Ah treatment showed a significant increase in the number and total duration of Np waves, as well as the time to first E1 and E2. Sh_Am treatment showed a significant increase in the number of C and Np waves as well as the total duration of Np, G, and Pd waves and the time to first E2, whereas the total duration of E2 was significantly shortened (**Supplementary Tables S2**, **S3**).

### 3.4 Effects of salt and alkali stress on aphid life-table parameters

Compared with the control and salt stress, alkali stress (whether separate alkali or mixed with salt treatments) significantly prolonged the duration of nymph aphids (*F*_7,232_ = 9.90, *P*< 0.001). High stress of alkali reduced the longevity of adult aphids (*F*_7,232_ = 2.21, *P* = 0.034). In addition, both salt and alkali stress significant shorter the reproductive period (*F*_7,232_ = 9.53, *P*< 0.001) and reduced the fecundity of adults (*F*_7,232_ = 17.65, *P*< 0.001) (**Table 1**).

**Table 1:** The development duration, longevity, and fecundity of *A. gossypii* on cotton seedlings of different concentrations of salt-alkali stress.

Treatment	Nymph period (days)	Adult longevity (days)	Reproductive days (days)	Fecundity
Control	4.93 ± 0.16c	25.73 ± 1.77ab	15.47 ± 1.13a	34.03 ± 2.91a
Salinity_middle	5.20 ± 0.23c	23.57 ± 1.24abc	10.37 ± 0.54cd	17.87 ± 1.22c
Salinity_high	5.30 ± 0.24c	22.53 ± 0.98bc	12.20 ± 0.78b	22.67 ± 1.86b
Alkalinity_middle	6.47 ± 0.21ab	25.87 ± 0.94a	11.30 ± 0.63bc	16.90 ± 1.33cd
Alkalinity_high	5.97 ± 0.17b	21.53 ± 1.16c	8.90 ± 0.63d	13.97 ± 1.11d
Sm_Am	7.43 ± 0.45a	25.30 ± 1.15ab	9.20 ± 0.35d	15.27 ± 0.74cd
Sm_Ah	6.97 ± 0.35a	21.67 ± 1.15c	9.80 ± 0.56d	16.53 ± 0.80cd
Sh_Am	6.57 ± 0.25ab	23.80 ± 1.01abc	9.13 ± 0.82d	13.93 ± 1.70d

Each value represents the mean of replications ± S.E. (n=30). For each parameter, ANOVA was used to compare the difference between different treatments. Multiple comparison was followed by Tukey’s HSD. Values followed with different lowercase letters showed significant difference among different treatments (P< 0.05).

Similarly, compared with the control and salt stress, alkali stress (whether separate alkali or mixed with salt treatments) significantly suppressed the aphid intrinsic rate of increase (*r*_m_) (*F*_7,16_ = 9.08, *P*< 0.001) and finite rate of increase (*λ*) (*F*_7,16_ = 9.12, *P*< 0.001). Both salt and alkali stress significantly reduced the net reproductive rate (*R*_0_) (*F*_7,16_ = 4.60, *P* = 0.006), and the stress of middle alkali lengthened the mean generation time (*T*) of aphid (*F*_7,16_ = 1.90, *P* = 0.135) (**Table 2**).

**Table 2:** The population growth parameters of *A. gossypii* on cotton seedlings of different concentrations of salt-alkali stress.

Treatment	*r*_m_	*λ*	*R*_0_	*T*
Control	0.32 ± 0.01a	1.38 ± 0.02a	29.17 ± 3.18a	10.59 ± 0.48bc
Salinity_middle	0.29 ± 0.02a	1.34 ± 0.02a	15.31 ± 1.47c	9.37 ± 0.30c
Salinity_high	0.30 ± 0.01a	1.35 ± 0.02a	19.43 ± 2.06b	9.96 ± 0.27c
Alkalinity_middle	0.22 ± 0.01bc	1.24 ± 0.01bc	14.49 ± 1.50cd	12.34 ± 0.43a
Alkalinity_high	0.23 ± 0.01b	1.26 ± 0.01b	11.97 ± 1.25d	10.57 ± 0.30bc
Sm_Am	0.21 ± 0.01bc	1.23 ± 0.01bc	13.09 ± 1.09cd	12.37 ± 0.52a
Sm_Ah	0.23 ± 0.01bc	1.26 ± 0.02bc	14.17 ± 1.19cd	11.64 ± 0.61ab
Sh_Am	0.20 ± 0.01c	1.22 ± 0.01c	11.94 ± 1.65d	12.65 ± 0.46a

Population growth parameters include intrinsic rate of increase (r_m_), finite rate of increase (λ), net reproductive rate (R_0_), and mean generation time (T). Each value represents the mean of replications ± S.E. (n=30). For each parameter, ANOVA was used to compare the difference between different treatments. Multiple comparison was followed by Tukey’s HSD. Values followed with different lowercase letters showed significant difference among different treatments (P< 0.05).

The results of two-sex life table analysis showed that salt or alkali stress significantly reduced the age-stage survival rate (*S_xj_*) of adults, whereas these treatments had no significant impact on aphid nymphs (**Supplementary Figure S1**). Moreover, salt or alkali stress also significantly reduced the age-specific survival rate (*l_x_*) and age-stage specific survival rate (*m_x_*) (**Supplementary Figure S2**).

### 3.5 Effects of salt or alkali stress on aphid population dynamics

**Figure 4:**
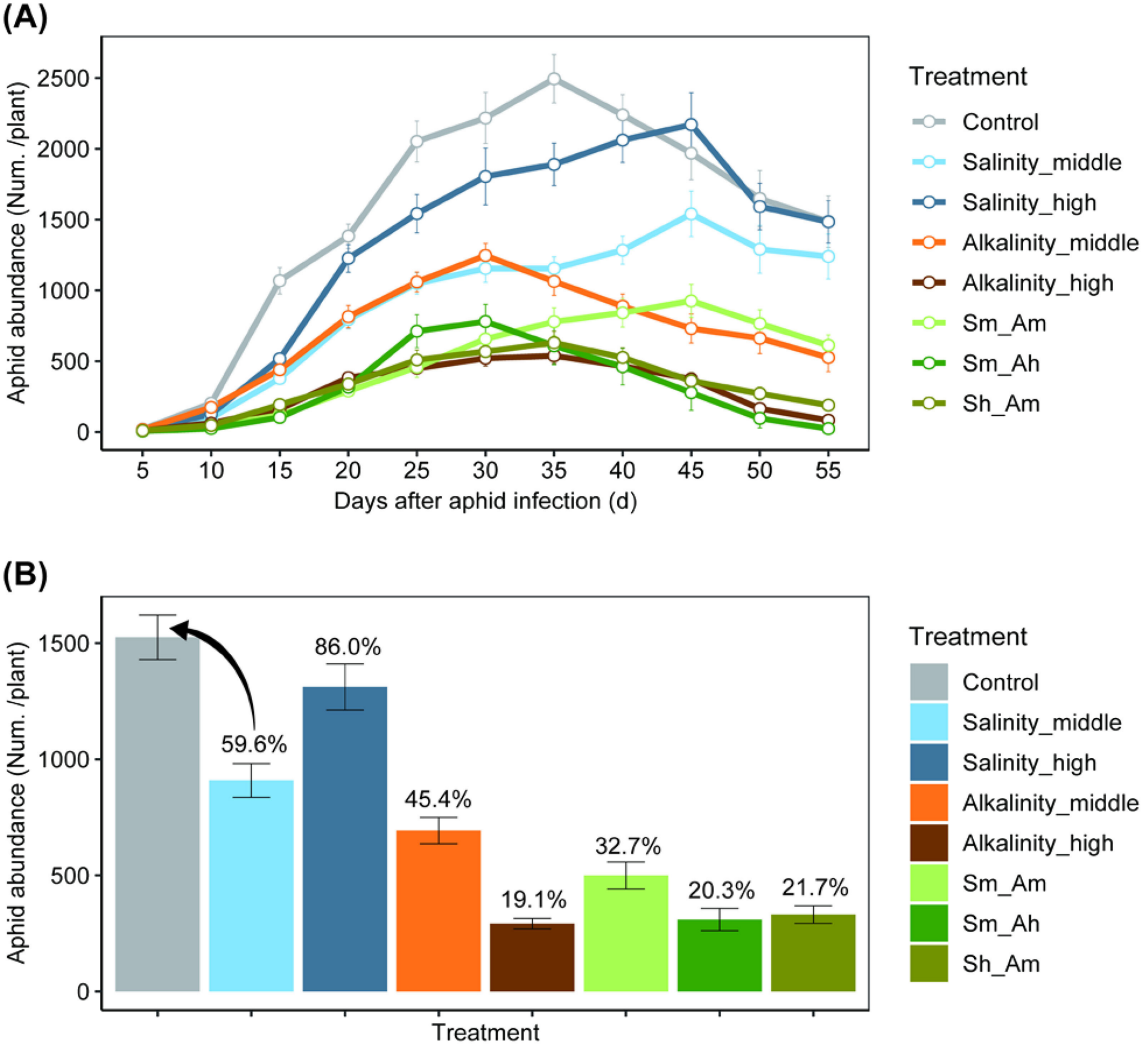
The population abundance of *Aphis gossypii* under different salt–alkali stress treatments. **(A)** shows aphid population dynamics; **(B)** shows the average abundance (numbers per plant) of aphids in different salt or alkali treatments. The percentage value above the error bars indicates the relative abundance of aphids in each treatment to the in relation to the control.

The population trends of *A. gossypii* were similar among treatments (**Figure 4A**; **Supplementary Table S5**). However, the population abundance did vary significantly among treatments (χ^2^ = 1459.19, df = 7, *P*< 0.001). With increasing salt or alkali concentrations, the number of *A. gossypii* on stressed cotton seedlings first increased and then decreased relative to the control on specific dates (χ^2^ = 1300.01, df = 10, *P*< 0.001). Aphid abundance in the control was significantly higher than in the other treatments after 15 days of salt or alkali stress. The peak abundance generally occurred during 30~45 days after aphid infestation (**Figure 4A**). The average abundance during the whole survey period was highest in the control, followed by the Salinity_high and Salinity_middle treatments, which were 86% and 59.6% as high as the control, respectively. Alkali stress also significantly reduced aphid abundance, which reached only 45.4% of the control value in the Alkalinity-middle treatment and 19.1% of the control in the Alkalinity-high treatment. For the combined saline-alkali treatments, aphid abundance was lower than either the saline treatments or the control (ranged of 20.3~32.7%) (**Figure 4B**).

### 3.6 PCA

**Figure 5:**
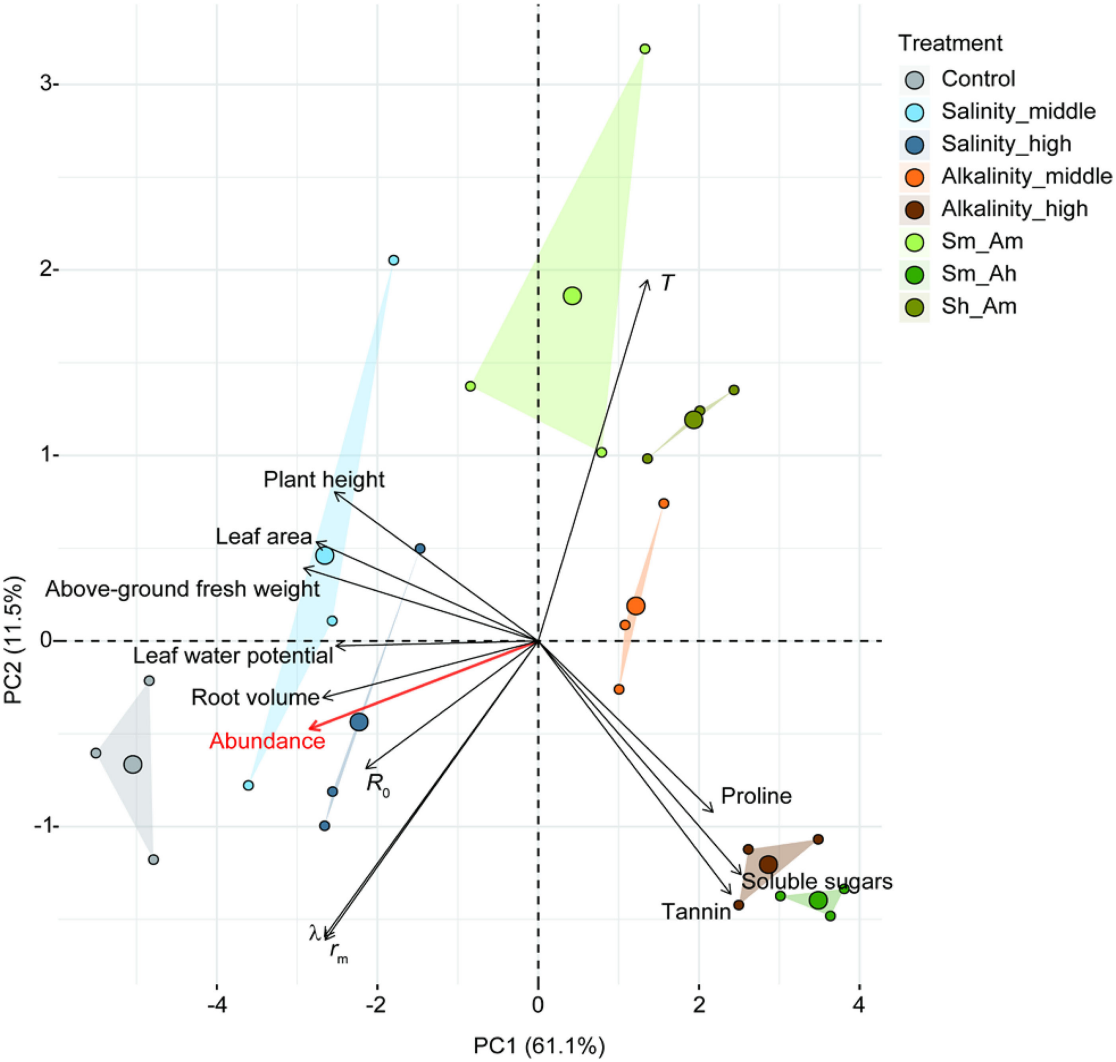
Principal component analysis (PCA) of salt–alkali stress-induced variation in plant growth traits, cotton physiochemical properties, life-table parameters of *A. gossypii*, and aphid abundance. Plant growth traits include plant height, leaf area, aboveground fresh weight, and root volume. The physiochemical properties include tannin, soluble sugars, proline, and leaf water potential. The life-table parameters include net reproductive rate (*R*_0_), intrinsic rate (*r*_m_), finite rate of increase (*λ*), and mean generation time (*T*). The red font represents aphid abundance. Triangles linked with sampling points indicated the group of different treatments, small circles represent the sampled points, and bigger circles in the central of triangles indicate the average position of sample points.

In PCA, the first two principal components axes (PC1 and PC2) explained a total of 72.6% of the variation of all principal components. The results suggested that these indicators, such as the growth, physiochemical characteristics of cotton plant, life-table parameters, and population abundance of the aphids, were well varied and classified under different salt or alkali stress treatments (**Figure 5**). The plant growth indices of leaf area, plant height, aboveground fresh weight, and root volume were highest in the Salinity-middle treatments and the control. The contents of tannin, soluble sugars, and proline levels in cotton leaves were higher in alkali stress or combined salt-alkali treatments than in the salinity stress and control groups, whereas leaf water potential showed the opposite trend. The individual life-table parameters, i.e., intrinsic rate of increase (*r*_m_), finite rate of increase (*λ*), and net reproductive rate (*R*_0_), as well as the population abundance (average value during the whole period), were all lower in the alkali or combined salt-alkali treatments than in salinity stress and control groups (**Figure 5**).

### 3.7 Cascaded effects of salt and alkali stress on plant growth and aphid density

Path analysis using SEM showed cascading effects of predictors on response variables. Specifically, plant growth, plant physiochemistry, and aphid population indices had different impacts on the aphid population abundance (**Figure 1**; **Supplementary Table S6**). Collectively, these predictors explained 44% of the variation (*R* ^2^ = 0.58) in aphid intrinsic rate of increase (*r*_m_) and 80% of the variation (*R* ^2^ = 0.80) in aphid population abundance. Salt or alkali stress-induced plant growth had a direct positive effect (the standardized path coefficient of this combinate variable was: *β* = 0.820, *P* = 0.004) on the aphid individual growth (*r*_m_) and directly (*β* = 0.544, *P* = 0.002) or indirectly affected the aphid population abundance. Individual indicators of plant growth, such as plant leaf area (*β* = 0.407, *P* = 0.012) and root volume (*β* = 0.535, *P* = 0.002), were significantly and positively related to aphid abundance. A high correlation (*β* = 0.838, *P*< 0.001) was found between plant growth and physiochemistry, which had a negative combinate effect (*β* = −0.068) on the aphid individual growth (*r*_m_). Also, the *r*_m_ value was significantly and negatively related to the level of tannin (*β* = −0.336, *P* = 0.047). In contrast, *r*_m_ value was positively related to leaf water potential (*β* = 0.553, *P* = 0.002). Moreover, high *r*_m_ values led to higher population abundance of aphids (*β* = 0.408, *P* = 0.013).

These results indicate that saline or alkali stress on cotton growth traits (leaf area and root volume) and its physiochemistry (water potential and tannin) significantly affected the intrinsic rate of increase (*r*_m_) of aphids, thereby affecting aphid population abundance.

## 4 Discussion

Saline or alkali stress from soil poses a serious threat to global agriculture (Penna et al., 2021; Liu et al., 2023a), by affecting growth and development of crops and their levels of key nutrient and metabolite substances, which in turn can alter the performance of pest herbivores (Dong et al., 2020). In our study, we used different concentrations of NaCl and Na_2_CO_3_ to simulate three types of stress (saline stress, alkali stress, and mixed stress) to determine the physiological and biochemical responses of cotton plants under different stress conditions. We found that with saline stress, the plant growth indices (i.e., plant height, leaf area, aboveground biomass, and root volume) of cotton seedlings showed a significant decline (**Figure 2**), consistent with previous studies (Quais et al., 2020; Duan et al., 2022). Compared with saline stress, we found that alkali stress suppressed the growth of cotton seedlings a greater degree. The severe inhibition of the root system caused by high pH resulted in a nutritional imbalance in cotton seedlings, leading to a sharp reduction in plant height (26.1%–38.3%), leaf area (58.3%–69.4%), aboveground biomass (69.6%–83.2%), and root volume (61.0–75.5). Our results agree with those of Peng et al. (2008), who found that the effects of alkalinity on survival of alfalfa seedlings was greater than those of salinity. The combination of salt and alkali stress can be more harmful to plant growth and development than the effects of either alone (Peng et al., 2008; Paz et al., 2014). We found that mixed stress had a strongly detrimental effects on plant growth. In terms of strength, the Sm_Ah (middle salt X high alkali) treatment was the most damaging, followed by Sh_Am (high salt X middle alkali), with Sm_Am (middle salt X middle alkali) being slightly weaker. However, our analysis also showed that some mixed stress treatments (Sm_Am and Sh_Am) did not have an increased impact on cotton plant growth in comparison with alkali stress alone. Thus, it is clear that mixed stresses do not merely sum the effects of individual salt or alkali stresses, but rather the plant response is more complex and varies with shifts in salt and alkali concentrations.

In terms of plant biochemistry, except for a significant decline in leaf water potential under the Salinity_high treatment, there were no obvious differences observed in other indices along the salt concentration gradient tested. However, as the alkali concentration increased, the levels of tannin, soluble sugars, and proline in cotton leaves all showed a significant upregulated trend, whereas leaf water potential showed a significant downward trend. In the case of mixed salt and alkali stresses, the water potential of cotton leaves experienced a significant decrease when compared with the control. The Sm_Ah treatment notably enhanced the levels of tannin, soluble sugars, and proline, having the highest values across all treatments. In the Sh_Am treatment, there was also a substantial increase in proline content. However, there was no significant difference in plant physiochemical characteristics between the Sm_Am treatment and the control. Our research findings indicate that salt and alkali stress exert varying degrees of inhibitory effects on the growth and development of cotton plants, and both alkali stress and mixed stress treatments further affected the nutrient and secondary metabolite levels in cotton plants. It was worth noting that the levels of tannin, soluble sugars, and proline under the Sm_Am and Sh_Am treatments in the mixed stress treatments were higher than those under salt stress alone but lower than levels under alkali stress (except for proline under the Sh_Am treatment). Previous studies have shown that under salt or alkali stress, plant cells synthesize and accumulate several small-molecule organic compounds, such as proline, soluble proteins, betaine, sugar, polyols, and polyamines, to maintain intracellular water potential (Shi et al., 2019). Proline is known as one of the most effective osmoprotectants in higher plants and is usually accumulated in large quantities under abiotic stress (Zanella et al., 2016; Launay et al., 2019; Li et al., 2022). A positive correlation has been documented between the accumulation of proline and improved stress tolerance in the plants (Elewa et al., 2017; Qirat et al., 2018; Sadeghipour, 2020). From this relationship, we infer that pH plays a key driver of the negative effects of mixed stress on cotton plants and that the presence of neutral salt ions to some extent can alleviate the effects of alkali stress.

Salt and alkali stress in our study had significant effects on the growth, development, and physiochemical aspects of plants, which subsequently plant features affecting herbivores, leading to notable changes in both individual aphid development rate and population size (Nam et al., 2017; dos Santos et al., 2021; Ma et al., 2021). Our study found that under salt stress, cotton aphids were primarily affected by a shortened reproductive period and a reduction in total fecundity, but that there was no effect on the nymphal developmental period or adult longevity. In contrast, alkali stress and mixed stress were more damaging to aphids. In addition to the shortened reproductive period and reduced fecundity, alkali or mixed stress also resulted in a longer nymphal developmental period and reduced adult longevity. Furthermore, compared with the control, salt and alkali stress treatments caused notable differences in aphid abundance (**Figures 4A, B**). According to SEM, the aphid individual growth rate (*r*_m_) was significantly and positively related to cotton growth traits (leaf area and root volume) and leaf water potential but was negatively to tannin levels. However, there was no significant change in tannin content under salt stress. We hypothesized that the primary reason for the decline in aphid fecundity due to changes in plant height and leaf area, leading to a decline in aphid abundance under stress. Throughout the experiment, we observed that as salt concentration increased, leaf area decreased, whereas leaf thickness increased; also, reduction in leaf water potential under high salt treatment resulted in insufficient cell turgor pressure, potentially impeding the feeding behavior of cotton aphids (Liu et al., 2023b). Our EPG results showed that under saline–alkali stress, the time of phloem ingestion decreased, whereas the time spent on non-probing of aphids increased (**Supplementary Tables S2**, **S3**). On the other hand, the times to first E1 and E2 were obviously increased under the combined stress treatments, which suggested that saline–alkali stress could affect the feeding behavior of cotton aphids by extending the time to locate feeding sites. Therefore, one of the reasons for the reduced fecundity of the aphid *A. gossypii* may be the decrease in the time the aphid spends ingesting phloem sap on plants exposed to saline–alkali stress. Tannin is well known as an important secondary metabolite of cotton that has significant negative effects on the survival and reproduction of pests, as well as their population densities (Ma et al., 2021; Qi et al., 2024). In this study, we found alkali stress significantly increased the level of tannin in cotton leaf. As a result, alkali stress and mixed treatments had lower aphid abundance than either the saline treatments and control.

In our study, we also found that the soluble sugar content of cotton leaves increased to varying degrees under alkali stress or mixed stress treatments. Soluble sugars can be utilized as nutrients by aphids and play a significant role in the growth, development, and reproduction of pests (Ryalls et al., 2014). According to Louda and Collinge (1992), excessive soluble sugars in plant tissues do not positively affect the ingestion of piercing-sucking insects, and indeed, Li et al. (2023) found that the densities of *Rhopalosiphum padi* (L.) and *Sitobion avenae* (Fabricius) was negatively correlated with levels of soluble sugars. We conclude that the increases of tannin and soluble sugars were important reasons for the reduction of the cotton aphid abundance. Our finding aid in understanding of the bottom-up interaction of different trophic levels on cotton under salt–alkali stress and help improve aphid integrated pest management (IPM) under conditions of global change.

## 5 Conclusion

This study indicated that salt or alkali stress significantly inhibited the growth of cotton plant (i.e., plant height, leaf area, and root volume), whereas alkali had a stronger effect on the levels of nutrients and secondary metabolites in cotton leaves, such as increased the content of soluble sugars, tannin, and proline, and decreased the leaf water potential. Moreover, that saline-alkali stress affected the feeding behavior of cotton aphids and reduced their fecundity and intrinsic rate of increase (*r_m_*), thereby indirectly causing a significant reduction in aphid population density.

## Data Availability

The original contributions presented in the study are included in the article/**Supplementary Material**. Further inquiries can be directed to the corresponding authors.
