# Supplementary material for: Effects of saline-alkali stress on cotton growth and physiochemical expression with cascading effects on aphid abundance
Source: Front Plant Sci. 2024 Oct 8;15:1459654. doi: 10.3389/fpls.2024.1459654 (PMC11493616; doi:10.3389/fpls.2024.1459654)

Supplementary Material

**Table S1.** The composition of different salinity and alkalinity treatments.

| Salt-alkali stress type | Treatment | Concentration |
| --- | --- | --- |
| No stress | control | Hoagland nutrient solution without NaCl and Na_2_CO_3_ |
|  |  |  |
| Salinity stress | Salinity_middle | 75 mM NaCl |
|  | Salinity_high | 150 mM NaCl |
|  |  |  |
| Alkalinity stress | Alkalinity_middle | 5 mM Na_2_CO_3_ |
|  | Alkalinity_high | 12 mM Na_2_CO_3_ |
|  |  |  |
| Mixed stress | Sm_Am | NaCl 75 mM+Na_2_CO_3_ 5 mM |
|  | Sm_Ah | NaCl 75 mM+Na_2_CO_3_ 12 mM |
|  | Sh_Am | NaCl 150 mM+Na_2_CO_3_ 5 mM |

**Table S2.** Comparison of the EPG parameters of *A. gossypii* under different salt-alkali stress.

| Treatment | Total # of C | Total # of E1 | Total # of E2 | Total # of np | Total time of np (min) | Total time of G (min) |
| --- | --- | --- | --- | --- | --- | --- |
| control | 169.87 ± 18.45cd | 30.80 ± 4.83cd | 27.80 ± 4.75cd | 0.27 ± 0.15b | 0.26 ± 0.14c | 4.14 ± 2.53b |
| Salinity_middle | 184.53 ± 22.01bcd | 67.07 ± 18.63abc | 64.60 ± 18.69abc | 0.27 ± 0.27b | 0.16 ± 0.16c | 4.79 ± 2.26b |
| Salinity_high | 270.00 ± 24.89a | 97.53 ± 16.83a | 91.40 ± 16.46a | 0.53 ± 0.29b | 0.20 ± 0.11c | 6.53 ± 4.50b |
| Alkalinity­_middle | 234.40 ± 16.35ab | 50.47 ± 11.00bcd | 47.53 ± 10.77bcd | 0.40 ± 0.40b | 1.01 ± 1.01c | 2.70 ± 1.93b |
| Alkalinity­_high | 217.20 ± 16.23abc | 74.93 ± 17.46ab | 71.13 ± 17.44ab | 0.47 ± 0.47b | 0.81 ± 0.81c | 5.23 ± 2.57b |
| Sm_Am | 241.40 ± 20.64ab | 34.93 ± 9.73cd | 25.60 ± 9.23d | 3.07 ± 1.18a | 4.97 ± 2.27bc | 6.59 ± 3.35b |
| Sm_Ah | 151.67 ± 23.35d | 15.73 ± 4.18d | 12.27 ± 4.04d | 3.47 ± 0.59a | 9.12 ± 2.98b | 9.52 ± 3.99b |
| Sh_Am | 241.00 ± 25.19ab | 48.40 ± 17.80bcd | 42.27 ± 17.68bcd | 3.60 ± 0.95a | 18.8 ± 5.88a | 30.74 ± 11.79a |

Note: Values represent the mean ± SE (n = 15). For each parameter, ANOVA was used to detect difference among treatments. Multiple comparisons among means were made using Tukey’s HSD. Values followed with different lowercase letters showed significant difference among different treatments (*P* < 0.05).

**Table S3.** Comparison of the EPG parameters of *A. gossypii* under different salt-alkali stress.

| Treatment | Total time of C  (min) | Total time of E1 (min) | Time to first E1 (min) | Total time of E2 (min) | Time to first E2 (min) | Total time of Pd (min) |
| --- | --- | --- | --- | --- | --- | --- |
| control | 202.65 ± 21.45bc | 53.70 ± 12.70ab | 0.92 ± 0.34b | 208.45 ± 22.94ab | 5.65 ± 2.56c | 10.44 ± 1.11c |
| Salinity_middle | 220.66 ± 21.92b | 83.07 ± 19.67a | 0.12 ± 0.03b | 160.54 ± 23.71bc | 10.35 ± 6.77c | 10.74 ± 1.35c |
| Salinity_high | 247.37 ± 21.56ab | 55.08 ± 8.32ab | 0.16 ± 0.10b | 152.15 ± 23.05bc | 1.78 ± 0.73c | 15.38 ± 1.36a |
| Alkalinity­_middle | 305.67 ± 26.48a | 43.69 ± 11.48b | 0.38 ± 0.13b | 112.40 ± 22.12c | 1.14 ± 0.40c | 14.52 ± 1.08ab |
| Alkalinity­_high | 234.05 ± 20.91b | 85.17 ± 15.74a | 1.20 ± 0.83b | 141.44 ± 20.57bc | 0.33 ± 0.07c | 13.03 ± 1.07ab |
| Sm_Am | 190.49 ± 15.48bc | 83.63 ± 11.32a | 2.09 ± 0.79ab | 175.40 ± 27.44bc | 17.89 ± 9.43bc | 15.45 ± 1.16a |
| Sm_Ah | 145.32 ± 22.44c | 49.26 ± 15.94ab | 3.49 ± 1.51a | 253.31 ± 30.13a | 53.80 ± 27.89b | 11.05 ± 1.76bc |
| Sh_Am | 235.63 ± 23.49b | 44.39 ± 8.79b | 1.17 ± 0.61b | 134.13 ± 31.29c | 134.13 ± 31.29a | 14.73 ± 1.44ab |

Note: Values represent the mean ± SE (n = 15). For each parameter, ANOVA was used to detect difference among treatments. Multiple comparisons among means were made using Tukey’s HSD. Values followed with different lowercase letters showed significant difference among different treatments (*P* < 0.05).

**Table S4.** The effect of salt-alkali stress on cotton growth (plant height, leaf area) based on GLMM analysis.

| Response variable | Fixed effect | χ^2^ | df | *P* |
| --- | --- | --- | --- | --- |
| plant height | Treatment | 419.40 | 7 | < 0.001 |
|  | Date | 729.40 | 5 | < 0.001 |
|  | Treatment: Date | 182.17 | 35 | < 0.001 |
| leaf area | Treatment | 366.41 | 7 | < 0.001 |
|  | Date | 80.06 | 5 | < 0.001 |
|  | Treatment: Date | 234.44 | 35 | < 0.001 |

**Table S5.** The effect of salt-alkali stress on aphid population dynamics based on GLMM analysis.

| Fixed effect | χ^2^ | df | *P* |
| --- | --- | --- | --- |
| Treatment | 1459.19 | 7 | < 0.001 |
| Date | 1300.01 | 10 | < 0.001 |
| Treatment: Date | 387.86 | 70 | < 0.001 |

**Table S6.** Path analysis with structural equation model (SEM) assessment of the causal relationships induced by saline-alkali stress on the cotton growth traits (leaf area, root volume) and physiochemical properties (water potential, tannin), the intrinsic increase rate (*r*_m_) of aphid individuals, and the aphid population abundance. Variables in path analysis were selected based on previous ANOVA, GLMM and PCA analyses after removing non-significant effects. Std. Estimate is the standardized path coefficient that indicates the effect size for each path. Asterisks represent significance levels (* *P* < 0.05, ** *P* < 0.01, *** *P* < 0.001). Goodness of model fit was evaluated using the Fisher’s C statistic, with *P* > 0.05 indicating the model was well fit by the data (Fisher’s C = 3.59, df = 2, *P* = 0.166). ~~ indicated the correlation between the composition variables of plant growth and their physiochemical properties.

| Response | Predictor | Estimate | Std. Error | DF | Critical Value | *P* | Std. Estimate |
| --- | --- | --- | --- | --- | --- | --- | --- |
| Population abundance | Intrinsic rate of increase (*r*_m_) | 5524.99 | 2041.97 | 21 | 2.71 | 0.013 | 0.408* |
| Population abundance | Plant growth | 0.64 | 0.18 | 21 | 3.61 | 0.002 | 0.544** |
| Intrinsic rate of increase (*r*_m_) | Plant physiochemical properties | 0.00 | 0.00 | 21 | -0.26 | 0.795 | -0.068 |
| Intrinsic rate of increase (*r*_m_) | Plant growth | 0.00 | 0.00 | 21 | 3.19 | 0.004 | 0.820** |
| ~~Plant physiochemical properties | ~~Plant growth | 0.84 | - | 22 | 7.19 | 0.000 | 0.838*** |

**Figure S1.** The difference of age-stage survival rate under different salt-alkali stress treatments.

**

**

**Figure S2.** The difference of age-specific survival rate and fecundity under different salt-alkali stress treatments.


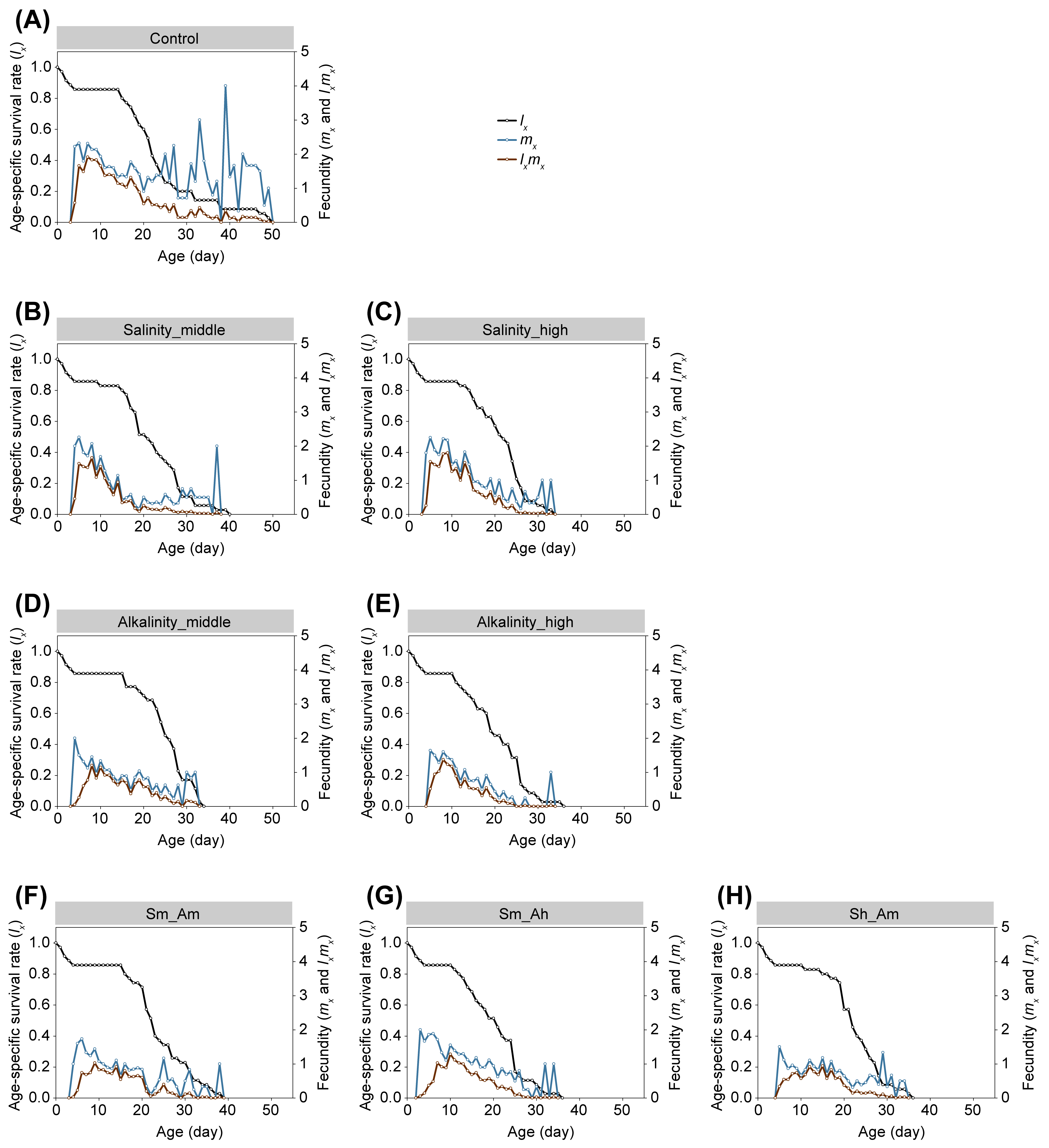

Supplement: Supplementary file 1 [file DataSheet1.docx]
